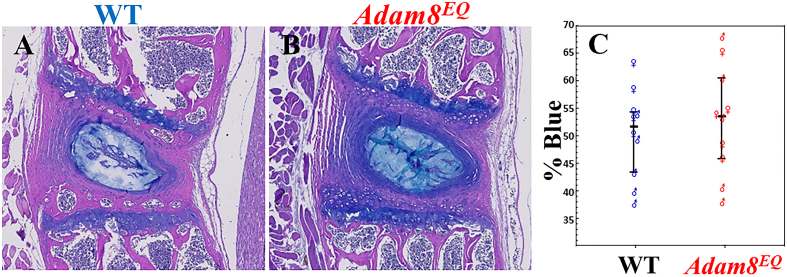# ADAM8 inactivation retards intervertebral disc degeneration in mice

**DOI:** 10.1016/j.gendis.2023.06.028

**Published:** 2023-08-02

**Authors:** Zuozhen Tian, Frances S. Shofer, Mingyue Fan, Alec Z. Sandroni, Lutian Yao, Lin Han, Ling Qin, Motomi Enomoto-Iwamoto, Yejia Zhang

**Affiliations:** a Department of Physical Medicine & Rehabilitation, Perelman School of Medicine, University of Pennsylvania, Philadelphia, PA 19146, USA; b Department of Emergency Medicine, Perelman School of Medicine, University of Pennsylvania, Philadelphia, PA 19104, USA; c Department of Orthopedic Surgery, Perelman School of Medicine, University of Pennsylvania, Philadelphia, PA 19104, USA; d School of Biomedical Engineering, Science & Health Systems, Drexel University, Philadelphia, PA 19104, USA; e Department of Orthopedics/Sports Medicine and Joint Surgery, the First Hospital of China Medical University, Shenyang, Liaoning 110001, China; f Department of Orthopedics, University of Maryland School of Medicine, Baltimore, MD 21201, USA; g Section of Rehabilitation Medicine, Corporal Michael J. Crescenz Veterans Affairs Medical Center, Philadelphia, PA 19104, USA

ADAM8 has been identified as the fibronectin-cleaving enzyme in human degenerative intervertebral disc (IVD) tissues.^1^ Increase of the active form of ADAM8 and its cleavage product, fibronectin fragment, correlated with an increased degree of IVD degeneration.^1^ Furthermore, fibronectin fragments have been shown to accelerate the progression of IVD degeneration.^2^ These findings suggest that ADAM8 may play a role in degenerative disc disease, a clinical problem with tremendous socioeconomic burdens in the United States.^3^

ADAM8 (aka MS2/CD156a), a membrane-anchored protein structurally related to snake venom disintegrins, is anchored to the plasma membrane and self-activates by shedding its prodomain. An ADAM8-global inactivation mouse model has been generated by introducing a point mutation, replacing the glutamic acid (E) at position 330 with glutamine (Q; abbreviated as *Adam8*^*EQ*^).^4^ The E to Q mutation prevents shedding of the prodomain, thereby preventing the self-activation of the enzyme. *Adam8*^*EQ*^ mice did not show any developmental defects and had milder collagen-induced arthritis than wild-type (WT) mice.^4^ Examination of the IVDs showed that young adult *Adam8*^*EQ*^ mice had more robust annulus fibrosus (AF) in histological structure compared with WT mice, but no striking differences in proteoglycan content.^5^ Since proteoglycan loss may progress with aging, the current study includes middle-aged mice.

**IVDs in the Adam8**^**EQ**^**mutant mice displayed higher amounts of proteoglycans.** At age 10 months, the lower lumbar spine of *Adam8*^*EQ*^ mice contained a higher proportion of proteoglycan-rich area than that of WT mice (Fig. 1A). Specifically, the proportion of Safranin O-stained area is higher in the IVDs of *Adam8*^*EQ*^ than in WT control mice (median with interquartile range/IQR = 22.7%, IQR: 4.3%–26.1% *vs*. 2.7%, IQR: 0.8%–14.0% respectively; *P* = 0.033). Additionally, among WT mice, female IVDs retained more proteoglycans than males (median = 9.0% *vs*. 1.3% respectively; *P* = 0.017). Similarly, among *Adam8*^*EQ*^ mice, female IVDs retained more proteoglycans than males (22.3% *vs*. 4.2%; *P* = 0.003; Fig. 1A').

**Figure 1:**
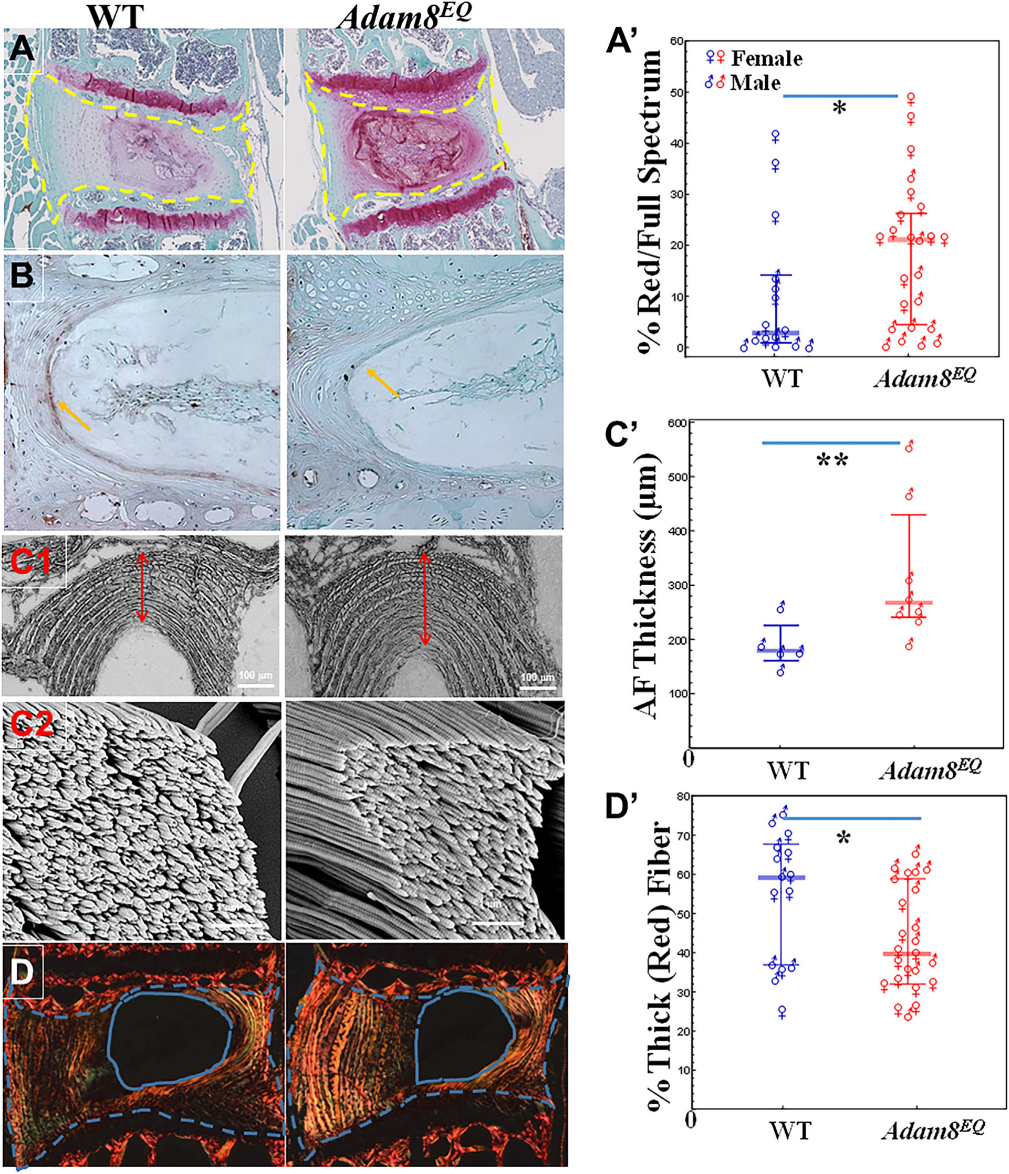
Comparisons between wild-type (WT) and mutant (*Adam8*^*EQ*^) mouse lumbar spine intervertebral discs (IVDs). **(A)** Representative images of the WT or *Adam8*^*EQ*^ mouse IVDs. Proteoglycans were stained red with Safranin O. The yellow outlines represent the areas quantified. **(A')** % proteoglycans stained red in all WT and *Adam8*^*EQ*^ mouse IVDs (15 WT mice [7 females ♀, 8 males ♂] versus 24 *Adam8*^*EQ*^ mice [13 ♀ and 11 ♂]). **(B)** Aggrecan degradation product (the neoepitope VDIPEN) immunostained. Arrows point to the innermost layer of the annulus fibrosus. **(C1)** Low magnification scanning electron microscopy images of AF. Scale bars, 100 μm. **(C2)** The high-magnification images showing collagen fibrils in the outer AF. Scale bars, 1 μm. **(C')** AF thickness (8 *Adam8*^*EQ*^ mice versus 5 WT male mice). **(D)** Discs stained with picrosirius red under a polarized microscope. The areas between blue dotted and solid lines are quantified. **(D')** Thick (red) collagen fiber pixel proportion in WT and *Adam8*^*EQ*^ mouse discs (15 WT mice [7 females ♀, 8 males ♂] versus 24 *Adam8*^*EQ*^ mice [13 ♀ and 11 ♂]). Each symbol represents one mouse. Error bars: median and interquartile range; ^∗^*P* ≤ 0.05, ^∗∗^*P* ≤ 0.01.

**Inactivation of ADAM8 function suppressed aggrecan degradation in IVD.** Aggrecan cleavage in lumbar IVDs in the WT and *Adam8*^*EQ*^ mice at 10 months of age was examined by immunostaining, using an antibody specific to the VDIPEN neoepitope. The WT IVDs contained products of cleaved aggrecan in the NP. Such phenotype was not detected in the *Adam8*^*EQ*^ IVDs (Fig. 1B), indicating that inactivation of ADAM8 function suppresses aggrecan degradation in IVD (4 mice per group). The reduced aggrecan degradation could explain, at least in part, the higher proteoglycan content in the IVDs of *Adam8*^*EQ*^ mice compared with WT controls.

**AF of the Adam8**^**EQ**^**mice was thicker than that of WT mice.** The mid-sagittal sections of lumbar IVDs were examined with scanning electron microscopy. The images revealed that the posterior AF was thinner in the WT controls than in *Adam8*^*EQ*^ mice (double-headed arrows in Fig. 1C1 indicate distance measured; median with IQR = 178.5, IQR: 178.1–191.1 μm in WT mice *vs*. 267.1, IQR: 243.7–390.9 μm in the *Adam8*^*EQ*^ mice; *P* = 0.019; Fig. 1C'). However, collagen fibril diameter did not show significant heterogeneity, although a larger sample number may reveal subtle differences in fibril size (Fig. 1C2). Taken together, these data suggest that there may be a larger number of collagen fibrils in the *Adam8*^*EQ*^ mouse IVD than in WT controls, resulting in thicker and more robust AFs.

**Adam8**^**EQ**^**lumber AF had a lower proportion of thick fibers than WT controls**. The mid-sagittal sections of the lower lumbar spine were stained with Picrosirius Red (PSR), and collagen fibers in the IVDs were examined under circularly polarized light (Fig. 1D). The color of collagen fibers (or fiber bundles) stained with PSR correlates with collagen thickness; as fiber thickness increases, the color changes progressively from green to yellow, orange, and red. PSR-stained collagen fibers in the low lumbar IVD were quantified, and pixel proportions of the red (thick), intermediate (orange and yellow), and thin (green) fibers were compared. *Adam8*^*EQ*^ mouse IVDs contained a lower proportion of large (red) fibers than those in the WT controls [median with IQR = 39.7%, IQR: 32.2%–58.2% in *Adam8*^*EQ*^ mice (*n* = 24) *vs*. median with IQR = 54.2%, IQR: 36.9%–67.7% in WT mice (*n* = 15); *P* = 0.05]. Additionally, among *Adam8*^*EQ*^ mice, male IVDs contained a higher proportion of red fibers than those of female mice (median = 56.8% *vs*. 35.1%; *P* = 0.015; Fig. 1D'). There were no striking differences in medium and thin (orange, yellow, and green) fibers between IVDs of mutant and WT mice (data not shown).

Previously, we have identified ADAM8 as a fibronectin-cleaving enzyme in human degenerative IVDs. ADAM8 active domain and its proteolytic product, a 29 kDa fibronectin N-terminal domain, are elevated in degenerative IVDs.^1^ These observations established its clinical relevance in disc degeneration. To examine the mechanistic aspects of ADAM8 in disc degeneration, a mouse line with a single nucleotide substitution, resulting in the replacement of the glutamic acid (E) at position 330 with glutamine (Q) has been acquired (*Adam8*^*EQ*^).^4^ This single amino acid substitution in the ADAM8 proteolytic domain prevents prodomain removal required for ADAM8 activation.^4^ Inflammatory marker gene expression was higher in injured *Adam8*^*EQ*^ mouse discs than in WT mice in response to a disc injury in the 3-month-old young adult mice, but no striking degenerative changes were found in either the WT or mutant mice of this age.^5^ In the current study, middle-aged (10-month-old) mice were used. The lumbar spine of *Adam8*^*EQ*^ mice retained more proteoglycan than their WT counterparts (Fig. 1A). This phenomenon has not been observed in young adult mice, likely because the loss of proteoglycans is progressive with aging. We have also shown reduced aggrecan degradation in the *Adam8*^*EQ*^ mouse IVD compared with WT controls, partially explaining the higher level of proteoglycans in the mutant mice. The AF of *Adam8*^*EQ*^ mouse IVD was thicker and appeared more robust than that of WT mice. Histological examination did reveal that AF of the *Adam8*^*EQ*^ mouse IVDs have fewer fissures between annular rings than AF of WT mice, in both the young adult^5^ and middle-aged mice. There is a smaller proportion of thick fiber bundles in the mutant mice than in the WT controls, as shown by PSR staining. Collagen fibers may also be thinner in the *Adam8*^*EQ*^ mice than in WT animals as shown by scanning electron microscopy, although a larger sample number is needed. The thicker AF and thinner collagen bundles/fibers suggest that there may be larger numbers of collagen fibers in the *Adam8*^*EQ*^ AF, consistent with an anabolic process. These findings are encouraging, in that ADAM8 may be categorized as a novel therapeutic target to conserve extracellular matrix and prevent disc degeneration. Molecular biological experiments to determine the mechanisms for better proteoglycan retention in the *Adam8*^*EQ*^ mice are worthy of further exploration.

In summary, we previously found that more ADAM8 correlated with increased degeneration in human IVD. A mouse line with ADAM8 proteolytic function inactivated (Adam8EQ) has been compared with WT mice. The Adam8EQ mouse retained more proteoglycans and had thicker AFs than WT controls. ADAM8 may be categorized as a novel therapeutic target to retard IVD degeneration.

## Ethics declaration

Animal use in this study was reviewed and approved by the Institutional Animal Care and Use Committee (IACUC) at the University of Pennsylvania, Philadelphia, PA, USA. All animal experimental procedures were carried out in compliance with the Animal Research: Reporting of *In Vivo* Experiments (ARRIVE) guidelines.

## Author contributions

All authors made a significant contribution to the work reported, whether that is in the conception, study design, execution, acquisition of data, analysis, and interpretation, or all these areas; took part in drafting, revising, or critically reviewing the article; gave final approval of the version to be published; have agreed on the journal to which the article has been submitted; and agree to be accountable for all aspects of the work. All authors read and approved the final submitted manuscript.

## Conflict of interests

The authors have no financial or other conflict of interests. None of the authors have any professional or financial affiliations that may be perceived to have biased the presentation.

## Funding

This work was supported, in part, by research grants to YZ from the 10.13039/100003202North American Spine Society (NASS) and Department of Veterans Affairs Healthcare Network (USA), and a grant from the 10.13039/100000069National Institute of Arthritis and Musculoskeletal and Skin Diseases (NIAMS; No. R21 AR071623). The histology core facility has been supported by a grant to the 10.13039/100016787Penn Center for Musculoskeletal Disorders (PCMD; No. P30AR069619).

## Figures and Tables

**figs1:**