# Supplementary material for: ADAM8 inactivation retards intervertebral disc degeneration in mice
Source: Genes Dis. 2023 Aug 2;11(3):101059. doi: 10.1016/j.gendis.2023.06.028 (PMC10825293; doi:10.1016/j.gendis.2023.06.028)
Supplement: Multimedia component 1 [file mmc1.docx]

**Supplementary materials and methods:**

***Mice.*** A breeding pair of *Adam8^EQ^* mice was gifted by Dr. AnneMarie Malfait. DBA/1LacJ mice (the Jackson Laboratory, Bar Harbor, ME, USA) were bred as WT controls. Mice were housed under pathogen-free conditions with environmental enrichment. A total of 47 mice (29 *Adam8^EQ^* and 19 WT mice), aged 39–40 weeks at the time of surgery, were used. Low lumbar spines including the lowest 2 motion segments were harvested *en bloc* from all mice for histological examinations.

***Histological evaluation.*** The lower lumber IVDs and portions of the adjacent bony vertebral bodies were isolated immediately after euthanasia, fixed with 4% paraformaldehyde, decalcified with 12.5% EDTA solution, and then embedded in paraffin and sectioned. The sections were subsequently stained with hematoxylin and eosin (H&E), 1% Alcian blue solution (Poly Scientific R&D Corp., Bay Shore, N.Y.; supplemental Fig. S1) or 0.1% safranin O solution (to reveal proteoglycan; Sigma; Fig. 1A), or 0.1% picrosirius red (PSR; to reveal collagen fibers; Sigma; Fig. 1D).

***Quantification of proteoglycan staining*.** Spine segments of *Adam8^EQ^* and WT mice, stained with safranin O, were digitized and analyzed by ImageJ software (NIH Image). The IVD regions were cropped (Fig. 1A, outlined in yellow) and converted to HSB Stack. The red color was defined as hue color 220–255 and 0–32. The pixel number of red and the entire color spectrum (hue color 0–255) were obtained for the lower lumbar IVD (L6/7), and the percentage of red in the entire color spectrum was calculated. Similarly, the digitized images of Alcian blue stained IVDs were processed using the ImageJ software, in order to quantify the blue staining. For each IVD, a region of interest (ROI) containing the anulus fibrosus and nucleus pulposus was selected. Each image was then converted to an HSB stack. The hue values were obtained from the histogram function and pasted into an Excel sheet. The blue component was measured from hue values 117 to 180 and the percentage was calculated as the ratio of blue pixels to total pixels of the ROI.

***Quantification of collagen fiber size on PSR-stained sections*.** The tissue sections, stained with PSR, were examined under a BXZ-700 microscope (Keyence, Itasca, IL, USA) with a circular polarizer filter and analyzed by ImageJ software (NIH Image). Briefly, the AF regions were cropped and converted to HSB Stack. We used the following hue definitions: red 2–9 and 230–256, orange 10–38, yellow 39–51, green 52–128. The pixel number of red, orange, yellow, and green (the colors of collagen fibers in order of decreasing thickness) were analyzed for the lower lumbar IVD (L6/7).

***Scanning electron microscopy.*** Paraffin sections were processed for proteoglycan removal, fixed and dehydrated, air-dried overnight, coated with a platinum-palladium mixture (a thickness of approximately 6 nm), and then imaged using a Supra 50 VP scanning electron microscope (Carl Zeiss, Germany). The AF thickness and collagen fibril diameters for the lower lumbar IVD (L6/7) were then quantified with ImageJ software (NIH Image).

***Statistics.*** Differences in safranin O staining (%red/full-color spectrum) and red, orange, yellow, and green colors in the PSR stained section between the WT and *Adam8^EQ^* mice, or male and female mice, were determined, where sex and mouse genotype were grouping factors. Due to the non-normality of safranin O and PSR staining data, Kruskal-Wallis tests were performed using genotype and sex as the grouping factors. To assess pairwise differences among the 4 genotype-sex groups, Dunn's test was used. For AF thickness, a Mann-Whitney test was performed because the data is non-parametric and only data on male mice were acquired. *P* values <0.05 were considered statistically significant. All analyses were performed using SAS statistical software (Version 9.4, SAS Institute, Cary, NC, USA). All figures were created using GraphPad Prism (Version 9.2, GraphPad Software. San Diego, CA, USA).

**Supplemental data:**

***Alcian blue staining of the mouse intervertebral discs (IVDs).*** At age 10 months, the lower lumbar spines of wild-type (WT) and *Adam8^EQ^* mice were stained with Alcian blue. The proportion of Alcian blue-stained area is not statistically different in the IVDs in the WT control mice from that in *Adam8^EQ^* mice (median with interquartile range (IQR) = 51.6%, IQR: 43.3%–54.4% *vs*. 53.5%, IQR: 45.8%–60.4% respectively; *P* = 0.55; Fig. S1).

**Supplemental Figure Legend:**

**Figure S1** Adam^EQ^ mouse lumbar spine intervertebral disc stained with Alcian blue. **(A, B)** Sagittal section of mouse lower lumbar spine motion segment. **(C)** % blue staining in wild-type (WT) and *Adam8^EQ^* mouse IVDs (10 WT mice [5 females ♀, 5 males ♂] and 10 *Adam8^EQ^* mice [5 ♀ and 5 ♂]). Each symbol represents one mouse.
